# Optimization of tablet processing as a reference material for microplastic detection methods

**DOI:** 10.1007/s00216-025-06271-7

**Published:** 2025-12-11

**Authors:** Mara Putzu, Yosri Wiesner, Christiane Weimann, Vasile-Dan Hodoroaba, Soledad Muniategui Lorenzo, Verónica Fernández-Gonzáles, Andy M. Booth, Amaia Igartua, Nizar Benismail, Laureen Coïc, Carine Chivas-Joly, Ivana Fenoglio, Andrea Mario Rossi, Andrea Mario Giovannozzi, Korinna Altmann

**Affiliations:** 1 Istituto Nazionale Di Ricerca Metrologica (INRiM), Strada Delle Cacce 91, 10135 Turin, Italy; 2 Università Di Torino (UniTO), Department of Chemistry, Via Pietro Giuria 7, 10125 Turin, Italy; 3 Bundesanstalt Für Materialforschung Und -Prüfung (BAM), Unter Den Eichen 87, 12205 Berlin, Germany; 4 Universidade da Coruña (UDC), Campus da Zapateira S/N, 15071 A Coruña, Spain; 5 SINTEF Ocean, Brattørkaia 17 C, 7010 Trondheim, Norway; 6 Nestlé Quality Assurance Centre (NQAC), Avenue Georges Clemenceau 1020, 88804 Vittel Cedex, France; 7 Laboratoire National de Métrologie Et d’essais (LNE), CARMEN Platform 29, Avenue Albert Bartholomé 23, 75015 Paris, France

**Keywords:** Analytical methods, Validation, Identification, Quantification, Plastic polymer, Polypropylene

## Abstract

Reference materials (RMs) are essential and highly demanded tools for the development and validation of methods for microplastic (MP) quantification in complex matrices, to ensure comparable and harmonized approaches aligned with EU commission criteria for monitoring MPs (e.g., Drinking Water Directive and Urban Wastewater Treatment Directive). This study investigates different approaches for optimizing the production of polypropylene (PP) RMs in the form of water-soluble tablets, which were carefully evaluated for their homogeneity and stability according to ISO Guide 30, ISO 33401, and ISO 33405. PP particles (1–100 μm) were produced by cryomilling and embedded in a lactose/PEG matrix, then pressed into tablets (18 µg theoretical PP mass). The production process was optimized by varying (i) the size distribution of the matrix components and (ii) the mixer instrument. The materials obtained were characterized by thermogravimetric analysis to assess the homogeneity distribution of MPs with respect to PP mass in the individual tablets and their stability over a 4-month period. The most promising approach, with a homogenous mass of 19 μg (standard deviation of 4 μg), relative standard deviation of 19%, was further investigated for homogeneity by comparison with thermo-analytical mass determination methods, such as TED-GC/MS (thermal extraction desorption-gas chromatography/mass spectrometry) and Py-GC/MS (pyrolysis-gas chromatography-mass spectrometry), and for number-based characterization using micro-Raman spectroscopy. Material characterization was also examined using laser diffraction, scanning electron microscopy, and ATR-FTIR. Based on the results, the optimized processing protocol yields a PP RM suitable for quality control and method performance studies supporting standardization.

**Supplementary Information:**

The online version contains supplementary material available at 10.1007/s00216-025-06271-7.

## Introduction

Microplastic (MP) contamination is gradually becoming a global problem [1]. These small plastic particles in the size range of 1–1000 µm [2] are mostly generated by the degradation of larger plastic exposed to environmental factors, such as wind abrasion, wave action, biodegradation, hydrolysis, and photodegradation by ultraviolet radiation from sunlight [3, 4]. MPs have been detected across all environmental compartments including marine, terrestrial, and atmospheric systems, as well as in biota [5–9]. They are ingested by consumers at various trophic levels, entering the human food chain and potentially impacting human health (e.g., intestinal damage, immune and reproductive problems, and neurotoxicity) [10, 11]. Reflecting the growing concern about MP, the 2018 EU Strategy for Plastics in a Circular Economy identified the need for more scientific research and solutions to address MP contamination, encouraging measures to reduce and prevent further pollution.

Efficient and reliable standardized measurement and monitoring methodologies are required in support of existing and proposed EU legislation, such as that proposed by the European Chemicals Agency (ECHA), Marine Strategy Framework Directive (MSFD), Urban Waste Water Treatment Directive (UWWTD), and Drinking Water Directive (DWD) [12]. However, the validation of standardized methods for robust and effective MP monitoring, including sample preparation and detection, remains an ongoing challenge, creating an urgent need for method development, as well as for (certified) reference materials (C(RMs)), to support methods validation, ensure comparability of measurement results, and provide reliable data for regulatory and research applications. The International Organization for Standardization (ISO) bodies ISO/TC 61/SC14/WG4 and ISO/TC 147/SC 2/JWG1 develop international guidelines for the sample preparation of MP in compost (ISO/CD 24899) [13] and the detection of MP in water. These standards include the identification and quantification of MP by number-based spectroscopy, such as micro Fourier–transform infrared spectroscopy (µ-FTIR) or micro-Raman spectroscopy (µ-Raman) (ISO/FDIS 16094-2) [14], and mass-based thermo-analytical methods (ISO/DIS 16094–3) [15], such as pyrolysis-gas chromatography-mass spectrometry (Py-GC/MS) and thermal extraction desorption-mass spectrometry (TED-GC/MS). Both techniques require (C(RMs)) to validate the analytical methods, assess their suitability for detecting plastic particles, and ensure that measurements—including both procedures and the resulting data—are comparable across MP studies.

According to ISO 33401 and ISO 33405, RMs are defined as being homogenous and stable in relation to one property of interest [16, 17], the certified value of which, for example mass, should be traceable to the International System of Units (SI). However, the preparation of RMs for MP analysis is challenging as they should mimic real MP found in environmental and food matrices in terms of their size, shape, surface functionality, and concentration (mass/number). Currently available RMs have been developed in the form of dissolvable soda tablets and soda capsules or salt cakes with a known number of added MP fragments or fibers [18–20]. Typical particle sizes in these materials are between 50 and 300 µm, which exclude smaller particles of higher relevance for health and environmental studies, limiting their applicability in the validation of measurement procedures.

The current work presents the preparation of an environmentally relevant polydisperse polypropylene (PP) reference material in the form of water-soluble tablets, containing particles predominantly in the 1–100 µm size range, intended for use with the most common and routine analytical tools for microplastic (MP) characterization, i.e., thermo-analytical and spectroscopic techniques. The primary aim is to optimize the processing protocol for producing a RM for use in method development and validation that has a particle size range (1–100 µm) that is relevant for human health exposure and risk assessment. The matrix is composed of lactose and polyethylene glycol (PEG 6000), which offers several advantages over soda tablets and soda capsules previously proposed as these materials are highly water-soluble, non-toxic, available in high purity, and suitable for direct compression into tablets. Importantly, lactose and PEG 6000 do not interfere with the signals from plastic during spectroscopic and thermo-analytical measurements. Five different approaches were tested for producing RMs, changing two variables in their preparation phase: (i) the particle size distribution of the matrix and (ii) the type of homogenization mixer instrument used, in which the tablet compounds were added step by step to achieve a homogeneous solid-phase dilution. PP was chosen as a suitable plastic polymer for producing MP RMs < 100 µm, due to its versatility and wide range of application across various industries, particularly in the packaging sector [21]. Tablets containing PP were characterized for homogeneity and stability according to mass with thermogravimetric analysis (TGA) and by complementary number-based approaches (µ-Raman, Raman microscopy) and mass-based approaches (Py-GC/MS, pyrolysis-gas chromatography/mass spectrometry, and TED-GC/MS, thermal extraction desorption-gas chromatography/mass spectrometry). Basic material characterization was conducted using laser diffraction (LD), scanning electron microscopy (SEM), and attenuated total reflection FTIR (ATR-FTIR).

## Materials and methods

All reported numerical values are presented as mean (standard deviation) of replicate measurements, with standard deviations (SD) indicated in parentheses. When appropriate, relative standard deviations (RSDs, %) are also specified.

### Materials

Ultrapure water obtained from a Milli-Q® IQ 7000 purification system (Merck Millipore, Germany), equipped with a 200-nm polyethersulfone filter to ensure particulate and bacteria-free water, was used for the dissolution and filtration of the tablet matrix and for cleaning procedures. PP pellets (Moplen RP320M, LyondellBasell) were used to prepare PP powder RMs. Directly compressible lactose (FlowLac90, D_50_, 138.2 (3.1) µm) was provided by MEGGLE GmbH & Co. KG. and PEG 6000 (D_50_, 98.5 (2.2) µm) was purchased from Merck. Ethanol absolute (99.9%, CHEMSOLUTE) was also used for various cleaning procedures.

### Production of reference material tablets containing PP microplastic

The production of RM tablets containing a PP MP involved a multi-step process that included preparation of the PP MP from pellets, mixing of the MP and matrix components, and compression into tablets. Considering the two variables, (i) the particle size distribution of the matrix and (ii) the homogenization mixer instrument, a set of five different tablet samples was prepared by pressing the PP MP RM with the size range 1–100 μm in a water-soluble matrix composed of lactose and PEG 6000. The PP MP was combined with either sieved or unsieved carrier matrix using three different mixer instruments, allowing the homogeneity and stability of the resulting RMs to be evaluated according to these parameters. The homogenization mixer instruments employed for tablet preparation included a Tumble mixer (LM-TM100, Laarmann, Roermond, Netherlands), VM Lab mixer (LFA machines), and Ball Mill (Retsch® CryoMill 100–240 V, 50/60 Hz). The preparation conditions for each sample are summarized in Table 1, and the mixing instruments used are shown in Figure S1.

**Table 1:** Preparation conditions for PP tablets

Sample	Composition	Matrix condition	Mixer type
Sample 1	PP (sieved) + matrix	Unsieved	Tumble mixer
Sample 2	PP (sieved) + matrix	Unsieved	VM Lab mixer
Sample 3	PP (sieved) + matrix	Sieved	Tumble mixer
Sample 4	PP (sieved) + matrix	Sieved	VM Lab mixer
Sample 5	PP (sieved) + matrix	Unsieved	Ball Mill

#### Preparation of PP microplastic and matrix components

MP particles were obtained by cryomilling of PP pellets using an Ultra Centrifugal Mill ZM200 (Retsch, Haan, Germany) with a rotation speed of 16,000 rpm. The mill was equipped with a cyclone. The cryogenic grinding of the pellets was conducted at low temperature using liquid nitrogen. A ring sieve with a 500-µm mesh size and a 12-teeth rotor was selected for the procedure. Before starting the milling process, the system was pre-cooled by adding approximately 60 mL of liquid nitrogen directly into the mill chamber. Meanwhile, the PP pellets were placed in a separate container and cooled with liquid nitrogen for a few minutes before being slowly introduced into the mill. The feeding process was carried out by alternating a maximum of five pellets, kept immersed in liquid nitrogen, with approximately 60 mL of only liquid nitrogen. The continuous addition of liquid nitrogen ensures that the rotor remains at a low temperature and prevents overheating, while limiting the number of pellets helps avoid the formation of aggregates that could interfere with the rotor movement. After the milling process, the resulting powder was transferred into a glass beaker, covered with aluminum foil to prevent contamination, and placed in the vacuum drying cabinet for at least 2 h (25 °C, 15 mbar).

To isolate MP particles in the 1–100 μm size range, the dried powder was fractionated with a dry-sieving method using a Retsch™ sieve shaker AS 200 Control (Retsch, Haan, Germany). Stacked stainless-steel sieves (20 cm in diameter) with mesh sizes of 500, 100, and 50 μm were shaken for 20 min at an amplitude of 1.40 mm and an interval time of 5 s. For each sieving cycle, the powder volume was set to 88 cm^3^, corresponding to the maximum capacity of the sieves. This volume was measured by carefully filling a graduated cylinder with 88 mL of powder, which is equivalent to 88 cm^3^ (1 mL = 1 cm^3^). The mass of powder for each cycle was then determined based on the measured volume and the density of each material, resulting in approximately 40 g for cryomilled PP powder, 60 g for lactose, and 54 g for PEG 6000. Minor variations in powder packing may occur during measurement, which could slightly affect the actual mass per cycle. Lactose (D_50_, 138.2 (3.1) µm) and PEG 6000 (D_50_, 98.5 (2.2) µm) powders were utilized as received from the supplier to produce three sets of tablets, while a separate batch of each matrix component was also sieved prior to use to isolate particles of a similar size range to the PP powder (1–100 µm). The sieving conditions for the matrix components were analogous to those applied to the PP. Due to differences in density, the maximum amount of material used in each sieving cycle was 60 g for lactose and 54 g for PEG 6000. Particles in the 1–100 µm size range were obtained by combining the yields from the 100–50 µm and < 50 µm mesh size. Additional information about the sieving process is available in the developed standard operating procedure (SOP) [22].

#### Mixing and compression into tablets

Tablets were produced by solid-phase dilution and the subsequent pressing of 200 tablets of each sample type (Table 1). The solid-phase dilutions were prepared with PP in the size range of 1–100 μm, which was mixed with lactose and PEG 6000 as the matrix components. Depending on the specific sample type, two different matrix conditions were applied: (i) lactose and PEG 6000 were used as received (unsieved) or (ii) lactose and PEG 6000 were sieved to the same size as the PP. Three different homogenization methods were then applied to the solid-phase dilution to assess their impact on the resulting PP content of the tablets: Tumble mixer, VM Lab mixer, and Ball Mill (Figure S1). Each combination of matrix condition and mixing instrument defined a specific sample, as previously listed.

Each individual tablet component was added into the homogenization mixer instrument and thoroughly mixed between different dilution steps to ensure homogeneous distribution of PP particles in the individual tablets. The sample preparation procedure for the Tumble mixer and the VM Lab mixer, which comprised 5 rounds of dilution and mixing, is summarized in Table S1. In the case of the VM Lab mixer, the mixing time for each step had to be extended, as the number of rotations of the mixing vessel was insufficient to ensure proper homogenization at the same time required for the Tumble mixer. For sample 5 (unsieved matrix, Ball Mill), the procedure was adapted to the specific requirements of the mixing instrument. Homogenization was carried out under liquid nitrogen, with a pre-cooling time of 10 min, six mixing cycles of 2 min each, and 2 min of cooling down during the individual cycles. A 50-mL grinding vessel with a single large and heavy ball of 25-mm diameter was used. The vessel was filled with 1/6 grinding ball, 1/6 sample, and 2/3 remaining free space, the latter required for ensuring ball movement and to account for possible sample expansion. Due to the limited volume of the grinding vessel, only 10 g of the solid-phase dilution could be used. Therefore, the quantities of the PP powder and matrix components were recalculated proportionally to match the 10 g target and directly added to the vessel to start the homogenization process. The sample preparation for Ball Mill is summarized in Table S2.

After each individual sample mixture (Table 1) had been produced, it was stored overnight in a vacuum drying cabinet (50 °C, 30 mbar) to remove any residual water prior to the tableting process. Tableting was performed with a TDP 5 Desktop Tablet Press (LFA Machines Oxford LTD, UK) operated in mode to ensure that the individual tablets were visually identical and had no breaking edges. To prevent the tablets from flaking, the stamp was cleaned at regular intervals with ultrapure water and ethanol, before being dried. Control weighing and solubility assessment were conducted in 10% of the tablets to ensure a constant tablet mass of 250 (5.0) mg and complete dissolution in water.

**Fig. 1:**
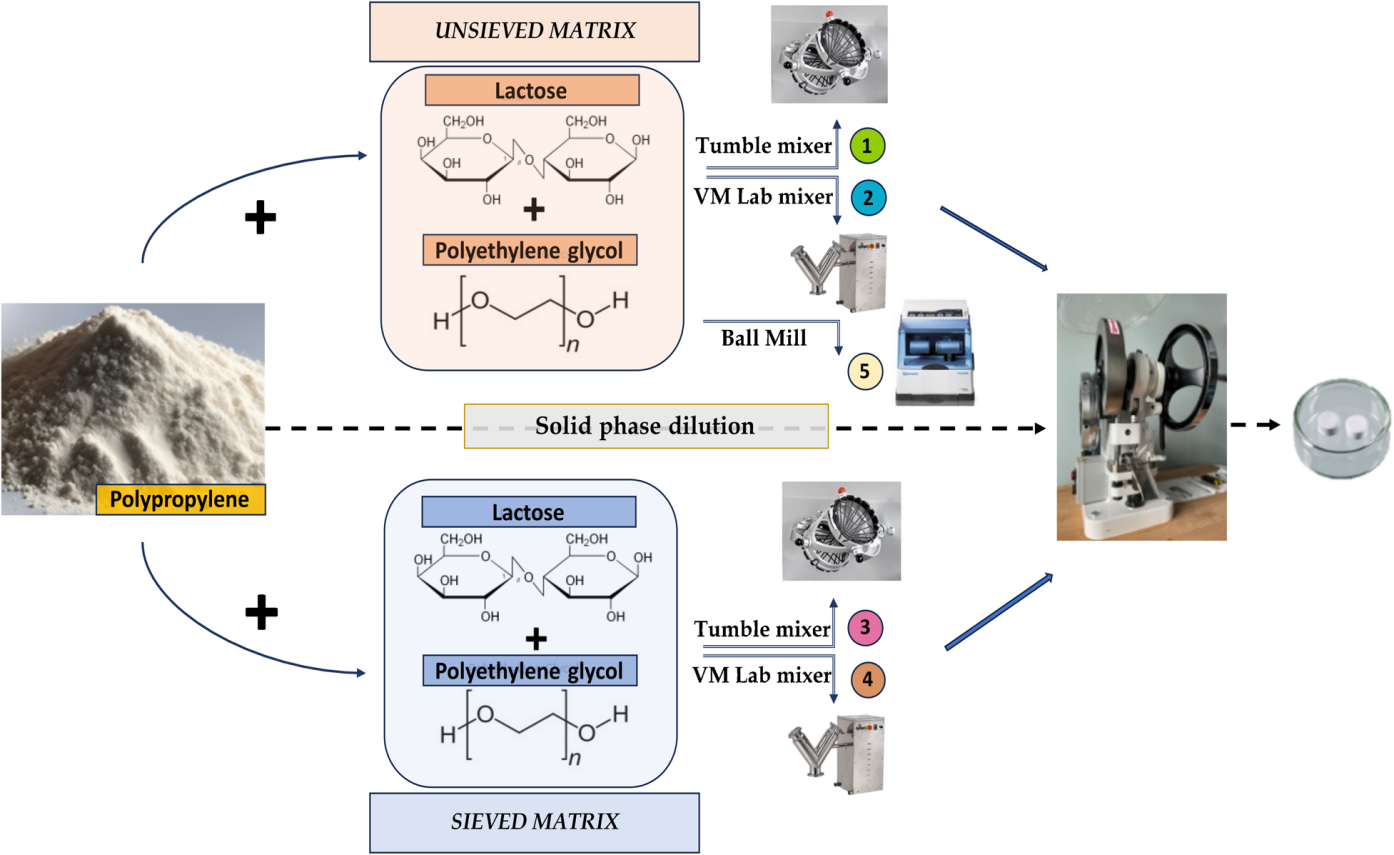
Production process for the PP microplastic reference materials

A comprehensive overview of the preparation of tablets is presented in Fig. 1.

The prepared PP RM tablets were subsequently analyzed using different analytical techniques by different laboratories and operators, each applying its own sample preparation workflows for the different techniques according to availability and the PP MP particle size fraction.

### Characterization of tablets and their individual components

#### Particle size measurement by laser diffraction

Particle size distribution measurements of the dry PP powder, lactose, and PEG 6000 (sieved and unsieved) were performed by LD using a HELOS BR instrument (Sympatec GmbH, Clausthal-Zellerfeld, Germany). The RODOS dry dispersion system with an injector width of 4 mm and a pre-pressure of 2.5 bar in combination with the micro dosing unit ASPIROS (transportation sleigh speed 100 mm/s) was applied. From 30 to 50 mg of powder was placed into a device-specific glass vial for analysis. The measurement time was 10 s, and 3 replicates were made with an R5 (4.5–875 µm) or R3 (0.9–175 µm) lens, according to the expected particle size range. All measurements were processed with the PAQXOS 4.1 software (Sympatec GmbH). Prior to the particle size measurements, the PP powder was weighed and passed through a deionizing device (Haug EN SL LC Generator combined with PRX U Frame Set, HAUG GmbH & Co. KG, Germany) to neutralize electrostatic charges on the particle surfaces. This treatment reduces agglomeration and improves the uniformity of dry dispersion within the laser diffraction system, thereby minimizing potential electrostatic effects on the measurement. Together with the use of three replicate analyses, this procedure contributed to obtaining consistent and reproducible particle size distributions, including the low size range.

#### ATR-FTIR

FTIR spectroscopy was performed using a Thermo Scientific™ Nicolet 8700 FTIR spectrometer in ATR mode to confirm the polymer composition of PP powder, ensure that no chemical or structural changes occurred during the production and sieving process, and guarantee unambiguous identification using other spectroscopic methods. The spectra were recorded from 500 to 4000 cm^−1^ with 32 scans and a resolution of 4 cm^−1^. The data obtained were processed with Omnic software, identifying the substances according to the spectral database.

#### Raman spectroscopy

Micro-Raman analysis was performed on PP MP particles collected on silicon (Si) filters after dissolving the PP tablets in water and subsequent filtration, using a LabRAM HR Evolution spectrometer (HORIBA France SAS). Each tablet was processed on different days by dissolving it directly in a glass funnel containing 1 L of reference water, previously analyzed and validated as free from MPs. At the end of the filtration, a solution of Triton™ X-100 (0.1% v/v in water, Thermo Fisher Scientific) was added to reduce particle agglomeration. Filtration was performed using Si filters with a pore size of 5 µm (macroporous silicon membrane, Smart membrane) to ensure effective retention of the majority of PP MP particles (1–100 µm) while maintaining an acceptable filtration flow rate. The filters were subsequently stored in closed Petri dishes until Raman analysis.

For each series of filtrations, a procedural blank was done with reference water prior to the filtration of PP tablets to evaluate the MP pollution that could occur during the filtration process.

All the manipulations were performed under a laminar flow hood. The laboratory adhered to the precautions outlined in [23], including hand washing, refraining from wearing makeup, and using cotton lab coats.

Spectra were acquired in the range of 550–2000 cm⁻^1^ using a 532-nm excitation laser with a power of 100 mW, an acquisition time of 0.1 s, and one accumulation. A 50× objective was employed for all measurements. Data acquisition was carried out with LabSpec software (v. 6.8.1.9), and particle identification was conducted using IDFinder software (v. 4.2, HORIBA France SAS). Spectra with a Hit Quality Index (HQI) > 70% were automatically assigned to PP, while those with 40% < HQI < 70% were manually verified.

#### Scanning electron microscopy

SEM was used to investigate the shape, surface morphology, and size of the particles. The PP, as well as sieved and unsieved lactose and PEG 6000 powder, was placed on double-sided adhesive carbon tape on the aluminum SEM stubs and coated with a 10-nm conductive gold layer to achieve surface electrical conductivity. High-resolution surface imaging of the particles was obtained using an EVO MA 10 SEM (Carl Zeiss Microscopy GmbH, Oberkochen, Germany) operated at an acceleration voltage of 10.00 kV and equipped with a tungsten cathode and a conventional secondary electron detector. Micrographs were captured at a resolution of 1024 × 768 pixels, a magnification range of 500×, and a working distance of 7 mm.

#### Thermogravimetric analysis

Ten tablets of each sample type were analyzed by TGA (Metter-Toledo TGA/DSC 3+) to assess the homogeneity and stability of the PP tablets. Samples were filtered through a specialized smart filter crucible (stainless-steel microfilter crucibles, 5 μm pore size, 9 mm in diameter, and 13 mm in height) (Gebr. Kufferath AG, GKD) [24] using approximately 50 mL of ultrapure water to dissolve and remove the tablet matrix. This innovative crucible design served a dual purpose: it functioned as a filtration device for collecting the PP MP particles from the dissolved tablet matrix and, critically, as an inert and thermally stable sample holder for subsequent thermal analyses, including both TGA and the hyphenated TED-GC/MS described later. After the filtration, the collected particles were stored in the oven at 50 °C for at least 3 h and later in the desiccator for another 2 h. Dried crucibles were weighed before and after filtration to calculate the mass of each sample. The dried loaded crucibles were placed in the TGA autosampler and heated from 25 to 600 °C [25] at 10 K/min under an inert atmosphere (N_2_, 30 mL). The decomposition temperature of polymers typically occurs between 350 and 500 °C [26]. The individual tablet components were also measured to determine their decomposition temperature range by measuring up to 1 mg of PP, lactose, or PEG 6000 separately in crucibles three times. The corresponding thermogravimetric mass loss and the corresponding first derivative curves were analyzed. Stability control was performed after 4 months on 7 samples.

#### Thermal extraction desorption-gas chromatography/mass spectrometry

Ten tablets from sample 3 were each analyzed by TED-GC/MS, which combines TGA (Mettler Toledo TGA 2 Star) with gas chromatography-mass spectrometry (GC/MS) (7890 B and 5977 B MSD, Agilent Technologies, Santa Clara, USA) [27]. Each tablet was first filtered through the smart filter crucible (as described in section “Thermogravimetric analysis”) and the collected PP MP particles in the dried crucibles are decomposed by TGA under a N_2_ atmosphere (from 25 to 600 °C), and the gaseous decomposition products are captured on a solid-phase sorbent, poly(dimethylsiloxane) (PDMS) (Sorb‑Star, ENVEA GmbH), for subsequent GC/MS analysis. A thermal desorption step was conducted by heating each sorbent to 200 °C, with the analytes subsequently cryo-focused on the cold injection system at −100 °C using liquid nitrogen. The decomposition products were then injected and separated by the GC column and analyzed in the MS. Additional information about a typical TED-GC/MS analysis is available in Nature Protocol [28]. PP was quantified using 2,4,6,8-tetramethylundec-10-ene as a marker (m/z 111).

#### Pyrolysis gas chromatography-mass spectrometry

Ten tablets from sample 3 were each analyzed by Py-GC/MS. PP particles from the tablets were isolated by filtration using a stainless-steel filter holder with a Whatman glass fiber filter (1.6 µm pore size, 13 mm filter size), following several validated protocols [29], and a 20-mL glass syringe connected to a VisiprepTM vacuum manifold (Supelco Sigma-Aldrich Co.). PP tablets were placed in the syringe barrel and half-filled with ultrapure water. After 4 min to allow dissolution, the sample was rinsed several times with additional ultrapure water, for a total volume of 150 mL, to ensure complete matrix removal. The filter was folded and placed in the pyrolysis ECO-cup (80 µL, Frontier Labs) and dried in an oven at 40 °C for a minimum of 4 h. Analysis was performed using a Frontier Multi-Shot Pyrolyzer (PY-3030D) (Frontier Labs, Japan) coupled with an Agilent 8860 GC and an Agilent 5977 A MSD (Py-GC/MS) (Santa Clara, CA, USA). The pyrolizer operated in single-shot mode with pyrolysis at 600 °C for 0.5 min. The pyrolizer interface and GC inlet temperatures were set to 300 °C, and the split ratio was 100:1. Helium was used as the carrier gas at a constant flow rate of 1 mL/min. Separation was achieved using an HP-5MS UI capillary column (30 m length, 0.25 μm film thickness, and 0.25 mm internal diameter). The column oven temperature was programmed to start at 40 °C for 2 min, then increased at a rate of 20°C/min up to 325 °C (14 min), followed by an increase of 30 °C up to 340°C. The transfer line temperature was maintained at 320 °C, the ion source temperature at 280 °C, and the quadrupole temperature at 150 °C. The ion source operated in full scan mode. PP was quantified using 2,4-dimethylhept-1-ene as a marker (m/z 70, 126) and 2,4,6,8-tetramethylundec-10-ene as a marker (m/z 69, 111) as confirmation markers, using external calibration.

## Results and discussion

### PP microplastic and matrix component yield

The sieving yields for each material per cycle are presented in Table 2, with values expressed as percentages for each mesh size range relative to the total amount added to the sieves. It is important to highlight that obtaining particles in the 1–100 µm size range is a time-consuming and labor-intensive procedure, with generally low yields observed for both the PP particles and the lactose and PEG 6000 matrix material, reflecting the low proportion of the target particles in the powders. This is particularly evident for PP, which exhibited an overall yield of approximately 1.5%, and for the lactose, which had a yield of 8% for particles 1–100 µm. Furthermore, residual material remained adhered to the mesh and to the sieving equipment following each cycle and could not be recovered, reducing the effective amount passing through the sieves and further limiting the quantity collected in each fraction.

**Table 2:** Size fraction yield of the cryomilled PP and the tablet matrix powder components

Mesh size (µm)	Polypropylene	Lactose	Polyethylene glycol
˃ 500	17.5%	0%	5.1%
500–100	71.2%	54.1%	59.6%
100–50	1.4%	7.7%	28.3%
< 50	0.1%	0.7%	0.6%
Total (%)	90.2	62.5	93.6
Mass loss (%)	9.8	37.5	6.4

### Characterization of tablet components

**Fig. 2:**
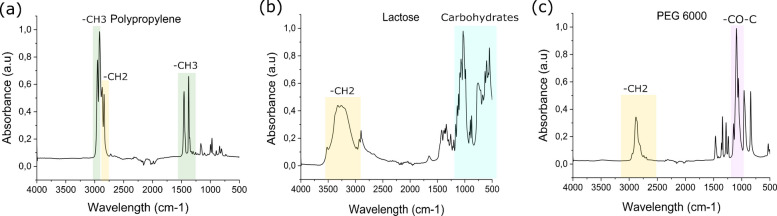
ATR-FTIR spectra of the three tablet components, PP (**a**), lactose (**b**), and PEG 6000 (**c**)

Figure 2a–c shows the ATR-FTIR spectra of the three tablet components. The spectrum for PP in Fig. 2a presents prominent peaks at 2960 and 2870 cm^−1^, as well as at 2930 cm⁻^1^ and 2850 cm⁻^1^, which are attributed to the asymmetric and symmetric stretching vibrations of methyl (–CH_3_) and methylene (–CH_2_) groups, respectively [30]. Additionally, peaks at 1455 and1375 cm^−1^ support the identification of the material as PP [31]. Lactose displays characteristic bands at 2900–3250 cm^−1^, corresponding to –CH_2_ asymmetric stretching, bands at 1140–1030 cm^−1^, corresponding to the intermolecular stretching of carbohydrates, and bands at 800–1000 cm^−1^, corresponding to carbohydrates, as shown in Fig. 2b [32]. In Fig. 2c, the spectrum for PEG 6000 exhibits a distinct absorption peak at 1097 cm^−1^, which is in the region of –CO–C stretching for ether groups, and -CH_2_ stretching vibrations in the region around 2900 cm^−1^, indicating the presence of methylene groups [33]. There was no obvious difference in the spectra for any of the materials before and after sieving. For PP, no changes in its chemical structure were observed either after the cryomilling, confirming that the production process does not affect the polymer’s functionality (Figure S2).

**Fig. 3:**
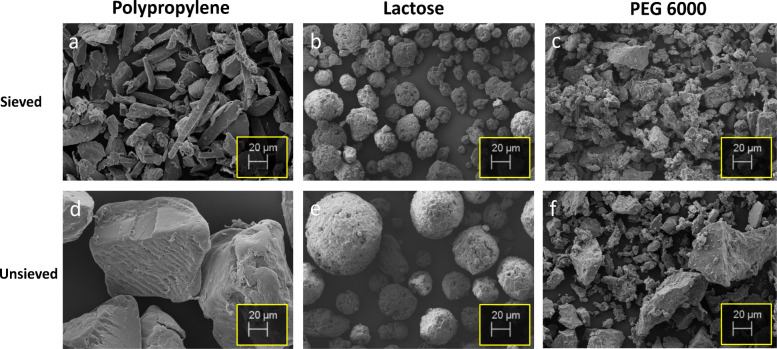
SEM images of sieved and unsieved PP, lactose, and PEG 6000

Following spectral confirmation by ATR-FTIR, the morphological properties of the three tablet components were characterized by SEM (Fig. 3), which confirmed that the PP and PEG 6000 are irregularly shaped, while the lactose particles were more spherical in nature. The sieved PP particles (Fig. 3a) exhibited a more elongated structure, which contrasts with the irregular-shaped fragments of the unsieved PP particles that had a lower aspect ratio (Fig. 3d**)**. The PEG 6000 particles presented as fragments with an irregular morphology in both sieved and unsieved samples (Fig. 3c and f). In contrast to the PP and PEG 6000 particles, the lactose particles had a much more rounded shape (Fig. 3b and e). All three particle types contained larger particles in the unsieved samples compared to the sieved samples. The variation in particle morphologies and size shown in the images supports the effectiveness of the sieving process for isolating a targeted size fraction.

**Fig. 4:**
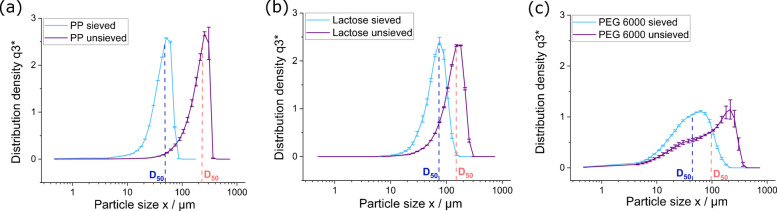
Particle size distribution via laser diffraction of PP (**a**), lactose (**b**), and PEG 6000 (**c**), with the D_50_ value reported in each graph

Figure 4a–c shows the particle size distribution of the three tablet components, sieved and unsieved, determined by LD. The unsieved PP, lactose, and PEG 6000 exhibited broad size distributions, where the distribution density values of D_50_ were 230.6 (6.7) µm, 149.1 (0.4) µm, and 99.2 (11.4) µm for PP, lactose, and PEG 6000, respectively. The D_50_ is the equivalent particle diameter of the measured volume below which 50.3% of the particles are. Considering the aim to produce PP MP RM in the 1–100 size range, the unsieved PP exhibited significantly larger particle sizes. After sieving, the particle size distributions were reduced, with D_50_ values of 48.4 (0.1) µm, 72.7 (1.5) µm, and 44.2 (1.0) µm for PP, lactose, and PEG 6000, respectively. The sieved PP particles were within the desired size range and comparable in size to the sieved matrix components, especially the PEG 6000. This variability in size among the unsieved tablet components may affect the homogeneity of solid-phase dilution during its preparation, which could increase the tablet-to-tablet uncertainty for the mass of each component, which should be minimized for RMs. The particle size distribution data indicates that pre-sieving the tablet components can help to increase the homogeneity of their distribution in the final tablets. Complete particle size distribution data, including D_10_ and D_90_ values, are provided in Table S3. The particle size distribution data indicate that the contribution of PP particles in the 1–10 µm range is negligible. Accordingly, for all subsequent analyses, filtration of the dissolved PP tablets using membranes with pore sizes larger than 5 µm was sufficient to retain the vast majority of PP particles, ensuring efficient sample handling without loss of relevant material.

### Homogeneity and stability control

**Fig. 5:**
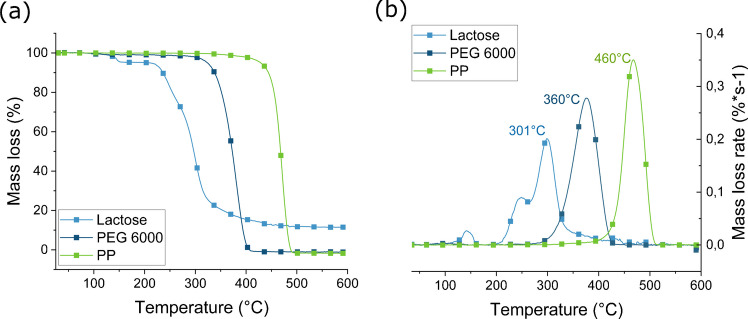
Mass loss (**a**) and mass loss rate (**b**) of PP, lactose, and PEG 6000

The mass loss curves from the TGA measurements and the corresponding first deviation curves, indicating the differential thermogravimetric (DTG) analysis with decomposition peak maxima of lactose, PEG 6000, and PP, are presented in Fig. 5. Lactose showed a mass loss between 200 and 300 °C, and PEG 6000 between 200 and 360 °C, which is consistent with available literature values for these materials [34, 35]. In contrast, PP showed a significant mass loss between 350 and 490 °C [36]. As lactose and PEG 6000 are water-soluble, they should be fully removed during the tablet filtration step and should no longer be present in the final sample. While the thermal degradation range of PEG 6000 partially overlaps with that of PP (around 350–360 °C), the observed mass loss starting from approximately 350 °C can be exclusively attributed to PP in samples that have been through the filtration step. This means that the mass of PP for each tablet can be calculated using the following equation:

1 $$\text{mass }\left(\text{PP in tablet}\right)=(\text{mass loss},350-490 ^\circ \mathrm{C})$$

**Fig. 6:**
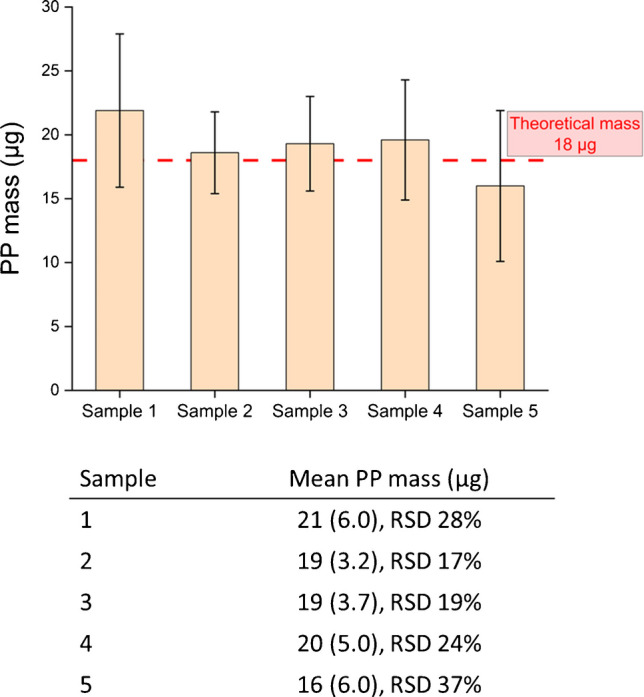
PP mass per tablet determined by TGA for each sample (*n* = 10), reported as mean PP mass (standard deviation, SD), with relative standard deviation, RSD%

The PP mass calculated for all five sample types produced (Table 1), with *n* = 10 tablets analyzed for each group, is presented in Fig. 6. The PP mass values across all five sample types ranged from 16 to 21 µg, with sample 1 exhibiting the highest calculated PP mass and sample 5 having the lowest PP mass. Considering that the theoretical PP mass within each tablet is 18 µg, the mean mass from *n* = 10 tablets for each set of samples calculated by TGA matched well with the theoretical value**.** However, a detailed analysis of the standard and relative deviations reveals differences in sample homogeneity, which are directly attributable to the preparation process. Sample 5, which was prepared using the Ball Mill and was unsieved, exhibited the highest variability, with a mean PP mass of 16 (6.0) µg, RSD 37%, rendering it the least suitable as RM. This variability is attributed to the grinding method and lack of sieving, which may have modified the particle size and morphology of the matrix components, making it harder to embed MP PP during the subsequent tableting process. In contrast, sample 2 (VM Lab mixer, unsieved) and sample 3 (Tumble mixer, sieved) appear to be the most promising as RMs, with a mean PP mass of 19 (3.2) µg, RSD 17%, and 19 (3.7) µg, RSD 19%, respectively. The strengths and limitations of these two samples are summarized in Table 3.

**Table 3:** Strengths and limitations of tableting processing for sample 2 and sample 3

Sample	Method	Strengths	Limitations
2	Unsieved matrices and VM Lab mixer	• No need to sieve the matrices makes the production process much faster	• Complex set up and handling; • Design for large quantities, requiring extended mixing time; • Tends to overheat, increasing the preparation time; • Difficult to remove the sample, with risk of loss • Time consuming; • Not commonly found in laboratories
3	Sieved matrices and Tumble mixer	• Suitable also for smaller quantities; • Reduced mixing times; • Easier set up and handling • No risk of sample loss; • More commonly found in laboratories	• Sieving of the matrices; • Time consuming to reach the desired amount of powder

**Fig. 7:**
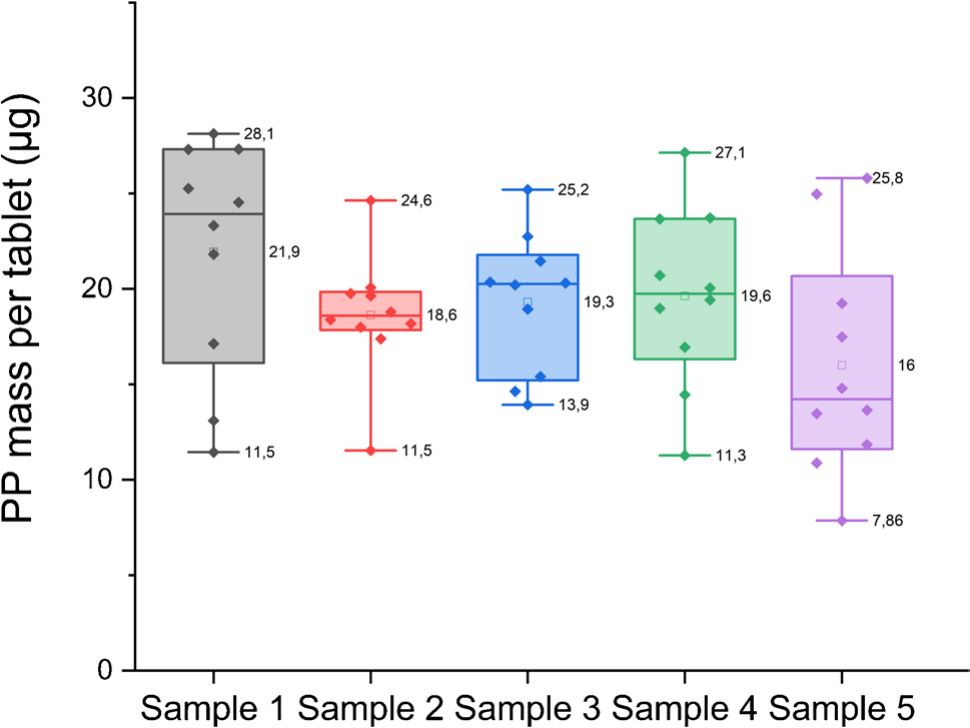
Distribution of PP mass across all five sample types

A one-way analysis of variance (ANOVA) was performed to assess the homogeneity of the five sample types, considering the effects of both matrix size distribution and homogenization mixer instrument. The analysis revealed no significant differences between the samples, with the *p*-values exceeding the commonly accepted threshold (*p* > 0.05). These results suggest that all five samples are statistically consistent across the production protocol, demonstrating the robustness of the different methods tested. The boxplot in Fig. 7 provides a clear representation of the distribution of data for each sample and highlights a critical distinction in uniformity between the most promising candidates. Sample 2 visually displays the most compact interquartile range (IQR), indicating the lowest dispersion for the central 50% of the data, and holds a slight advantage in overall variability with an RSD of 17% compared to RSD 19% for sample 3. Both these RSDs must be contextualized within the current state-of-the-art metrology for MPs. While these values do not fully meet the homogeneity requirements for a fully certified RM intended for quantification with low uncertainty, according to ISO 33405:2024 [17], they are consistent with the process variability reported by major metrological institutions for similar materials which report RSDs up to 15.8% during its in-process mass-based controls [37].

However, despite the marginal statistical superiority of sample 2, sample 3 was considered the most suitable candidate RM due to its superior robustness and control over extreme values across the entire distribution. While sample 2’s IQR is tighter, its minimum value (11.5 µg) and maximum value (24.6 µg) result in a wider absolute range (13.1 µg). In contrast, sample 3 with values ranging from 13.9 µg to 25.2 µg, exhibits a narrower overall range of 11.3 µg. This lower overall data spread for sample 3 is a crucial quality indicator for RM [38]. Considering this finding, combined with the favorable TGA results and the practical advantages in terms of preparation, reproducibility, and robustness of the manufacturing process, sample 3 was ultimately selected as the most suitable candidate RM.

**Fig. 8:**
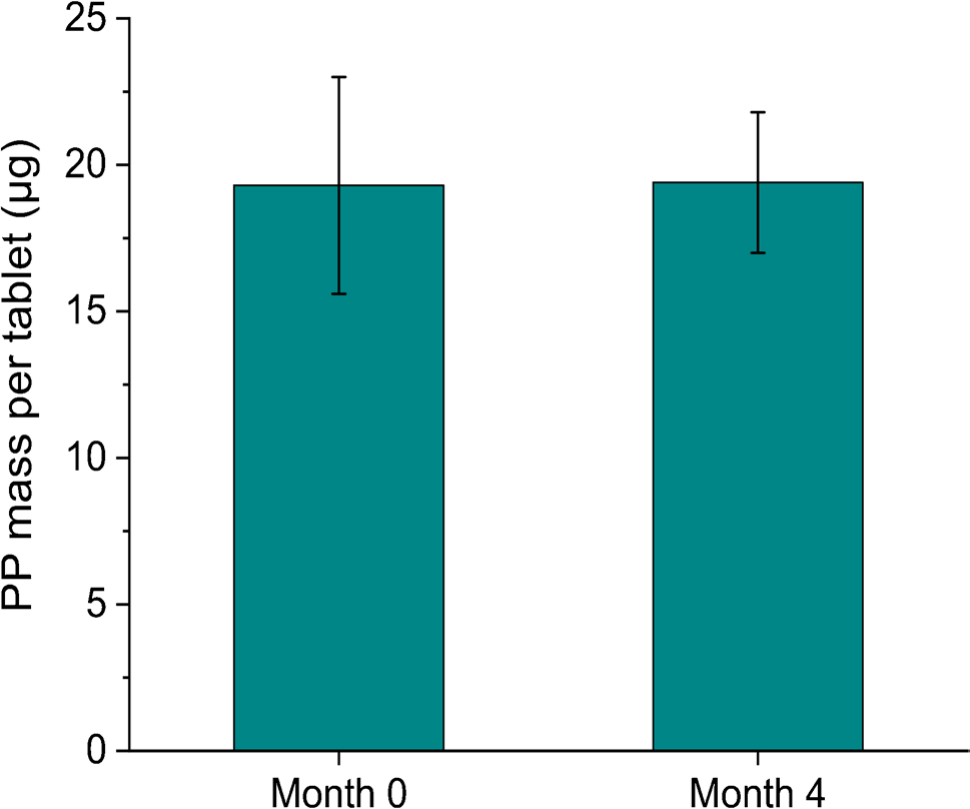
PP masses per tablet determined by TGA as stability control assessment

Stability control assessment of the PP tablets was performed on 7 tablets from sample 3 after 4 months by TGA (Fig. 8). No significant mass variations were observed in the samples after 4 months (19.4 (2.4) µg, RSD 12%) compared to the values obtained at month 0 (19.4 (3.7) µg, RSD 19%). One-way ANOVA confirmed no significant differences between the two time points (Fig. 4S), with the *p*-value exceeding the commonly accepted threshold (*p* > 0.05), indicating that the PP tablets maintain mass stability over time.

### Mass and number-based quantification

**Fig. 9:**
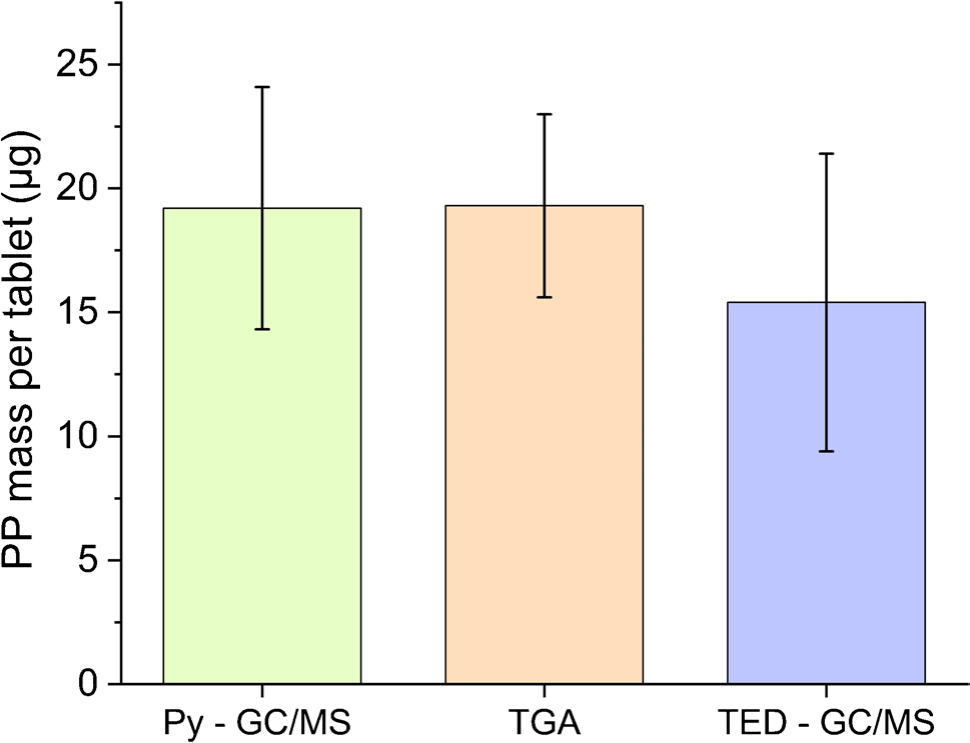
PP masses for sample 3 determined by mass-based methods (TED-GC/MS and Py-GC/MS) across two laboratories (*n* = 10)

Sample 3, identified as the most suitable as an RM, was further analyzed with additional mass-based methods to quantify the PP mass present in the tablets to support the TGA data. A total of 20 tablets were characterized in two different laboratories by TED-GC/MS (1 lab) and Py-GC/MS (2 lab). The two methods yielded comparable results, with a calculated mean mass of 15 (6.0) µg, RSD 39% (Lab 1) for TED-GC/MS, a mean mass of 19 (5.0) µg, RSD 25% for Py-GC/MS (Lab 2) (Fig. 9). Both techniques provided values within the range of the theoretical tablet mass and consistent with those obtained from homogeneity control via TGA (19 (4.0) µg, RSD 19%). The slightly higher RSD observed for TED-GC/MS (RSD 39%) compared with Py-GC/MS (RSD 25%) is likely primarily due to the analytical procedure, although a small contribution from variability between PP tablets cannot be ruled out. All 20 tablets were prepared from the same material and were homogeneous according to TGA results, indicating that sample-related variability is expected to be minimal. Py-GC/MS provides complete thermal decomposition of the polymer into a reproducible pattern of fragments, which tends to standardize the analytical signal and thus reduce variability. In contrast, TED-GC/MS relies on thermal desorption, a process inherently more sensitive to subtle variations in the physical state or morphology of the sample, which can lead to a less reproducible signal. As both methods are destructive, only a single measurement can be performed per PP tablet, preventing direct assessment of repeatability on the same sample. To minimize sample-related variability, 10 independent PP tablets of the same sample were analyzed in each laboratory. Despite these limitations, both methods provide comparable quantification of PP across independent tablets, as further supported by a one-way ANOVA, which revealed no statistically significant differences among the three methods (TGA, TED-GC/MS, and Py-GC/MS) (*p* > 0.05; Fig. 4S), confirming the suitability of the PP candidate for method performance studies aimed at assessing analytical precision. However, method-specific optimization would still be needed to fully reduce analytical variability and assess analytical precision.

**Fig. 10:**
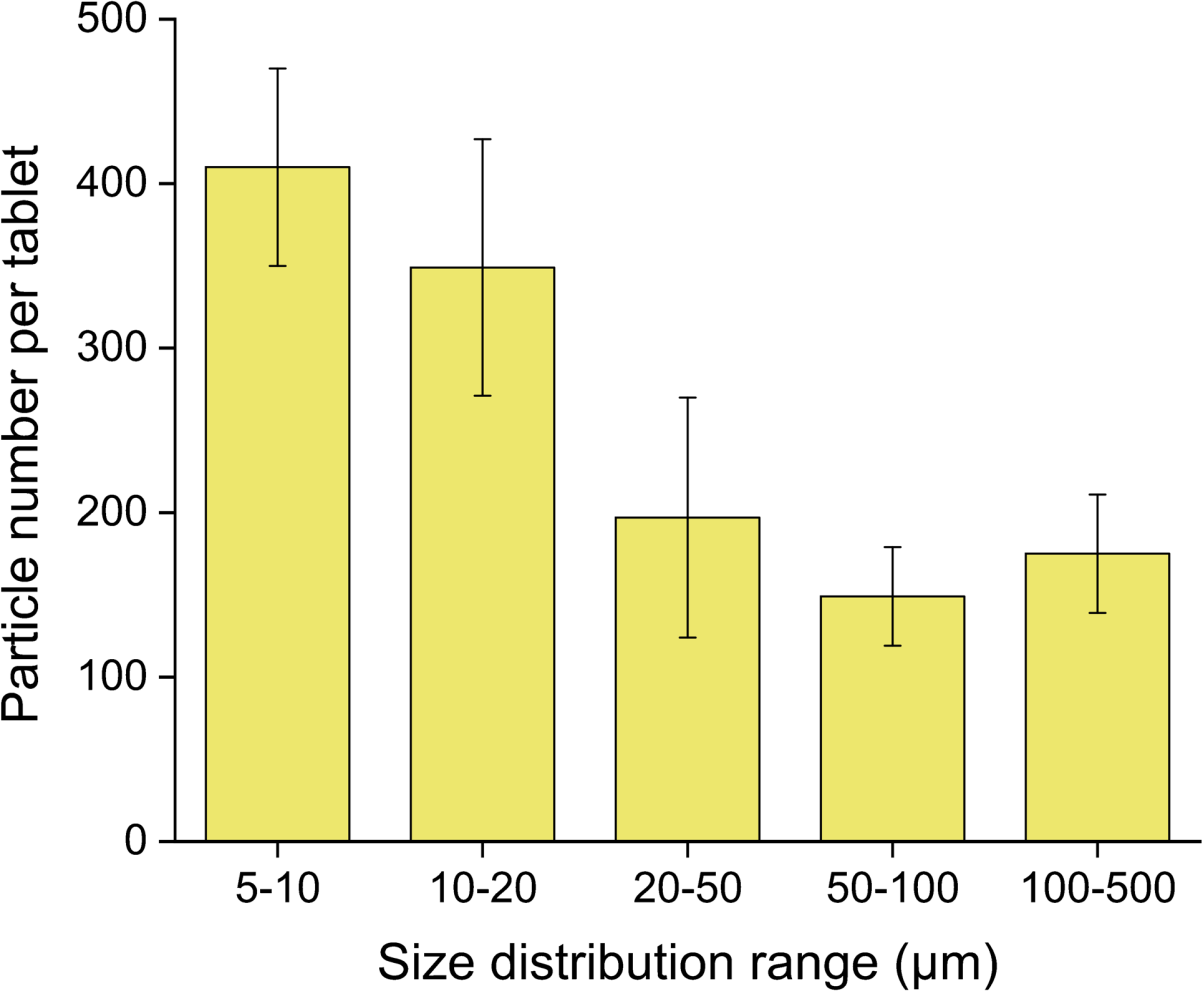
PP particle number detected by spectroscopic method

To complement the mass-based analysis, the PP tablets were further analyzed for particle number and size distribution using µ-Raman (Fig. 10). A total of 10 tablets were analyzed individually, with the PP particles present in each sample being quantified and categorized into the following size ranges: 1–5, 5–10, 10–20, 20–50, 50–100, 100–500 µm. The values were compared to those determined by LD for the pristine PP particles. A mean total PP particle number of 1284 (240.0), RSD 19% per tablet was detected; the spatial distribution of these particles on the Si filter surface is shown in Fig. S5. In addition to PP, other polymer types, such as polystyrene (PS) and polyethylene terephthalate (PET), were also detected on the sample filter (Table S4). Some of these were also present in the procedural blanks and are therefore attributed to background contamination during sample processing (e.g., filtration), while others were found exclusively on the sample filters, likely introduced during tablet preparation. In both cases, the contamination levels were low, with MP particle size mainly in the 5–10 µm range. The corresponding results for procedural blanks are reported in Table S5. No blank subtraction was applied, as the PP background levels were considered negligible (3(1.0) particles) relative to the total particle numbers observed in the tablet samples, confirming that the PP particles detected in the sample filters originate exclusively from the PP RM tablets. While LD analysis of pristine PP particles indicated that 50% of the sample was smaller than 48.4 (0.1) µm (Fig. 4), µ-Raman revealed a higher number of particles in the lower size ranges, 5–10 and 10–20 µm, which together accounted for approximately 60% of the total particle number. The µ-Raman analysis also detected particles in the 20–50 and 50–100 µm ranges, meaning that approximately 90% of the total PP particle number in the tablets was below 100 µm. Despite their different detection principles, LD and µ-Raman provide broadly comparable results in terms of the particle size distribution of PP present in the tablets.

## Conclusions

The lack of certified reference materials (RMs) has long hindered the validation and harmonization of analytical approaches for microplastics (MPs) in environmental and food matrices, including both sample preparation and instrumental methods. This study demonstrates the successful optimization of a processing protocol for producing PP tablets as feasibility-stage candidate RMs for MP particles by optimizing key factors such as the particle size distribution of the matrix components used to encapsulate the MP particles and the mixing instruments employed to ensure the required robustness. The suitability and comparability of these PP tablets for method assessment in MP identification and quantification have been demonstrated across multiple analytical techniques, in alignment with existing European directives. Complementary evidence from TGA, TED-GC/MS, Py-GC/MS, and Raman spectroscopy confirms their statistical consistency, stability, and realistic polydisperse particle distributions, reflecting environmental MP heterogeneity. Minor variability between PP tablets and techniques is expected but does not compromise their use for improving method evaluation, according to the current metrological challenges in MP analysis.

While this work focuses on PP, the approach is transferable to other polymers, enabling the production of well-characterized, fit-for-purpose RMs to support microplastic research and reliable monitoring in environmental and food matrices.

Overall, this work represents an important step toward bridging the gap between research and routine analysis, supporting regulatory and scientific efforts to advance the standardization and harmonization of microplastic methodologies.

## Supplementary Information

Below is the link to the electronic supplementary material.

Supplementary Material 1 (DOCX 1.85 MB)

## Data Availability

Data is available upon reasonable request from the corresponding author.
