# Supplementary material for: Optimization of tablet processing as a reference material for microplastic detection methods
Source: Anal Bioanal Chem. 2025 Dec 11;418(4):1001–14. doi: 10.1007/s00216-025-06271-7 (PMC12901254; doi:10.1007/s00216-025-06271-7)
Supplement: Supplementary file 1 — Supplementary Material 1 (DOCX 1.85 MB) [file 216_2025_6271_MOESM1_ESM.docx]

SUPPLEMENTARY INFORMATION (SI)

**Optimization of tablet processing as a reference material for microplastic detection methods**

Mara Putzu^1,2^, Yosri Wiesner^3^, Christiane Weimann^3^, Vasile Dan Hodoroaba^3^, Soledad Muniategui Lorenzo^4^, Verónica Fernández-Gonzáles^4^, Andy M. Booth^5^, Amaia Igartua^5^, Nizar Benismail^6^, Laureen Coïc^6^, Carine Chivas-Joly^7^, Ivana Fenoglio^2^, Andrea Mario Rossi^1^, Andrea Mario Giovannozzi^1^, Korinna Altmann^3^

^1^ Istituto Nazionale di Ricerca Metrologica (INRiM), Strada delle Cacce 91, 10135 Turin, Italy

^2^ Università di Torino (UniTO), Department of Chemistry, Via Pietro Giuria 7, 10125 Turin, Italy

^3^ Bundesanstalt für Materialforschung und -prüfung (BAM), Unter den Eichen 87, 12205 Berlin, Germany;

^4^ Universidade da Coruña (UDC), Campus da Zapateira s/n, 15071 A Coruña, Spain

^5^ SINTEF Ocean, Brattørkaia 17 C, 7010 Trondheim, Norway

^6^ Nestlé Quality Assurance Centre (NQAC), Avenue Georges Clemenceau 1020, 88804 Vittel Cedex, France

^7^ Laboratoire national de métrologie et d'essais (LNE), CARMEN Platform 29, Avenue Albert Bartholomé 23, 75015 Paris, France

Corresponding author: Korinna Altmann, e-mail: [korinna.altmann@bam.de](mailto:korinna.altmann@bam.de)


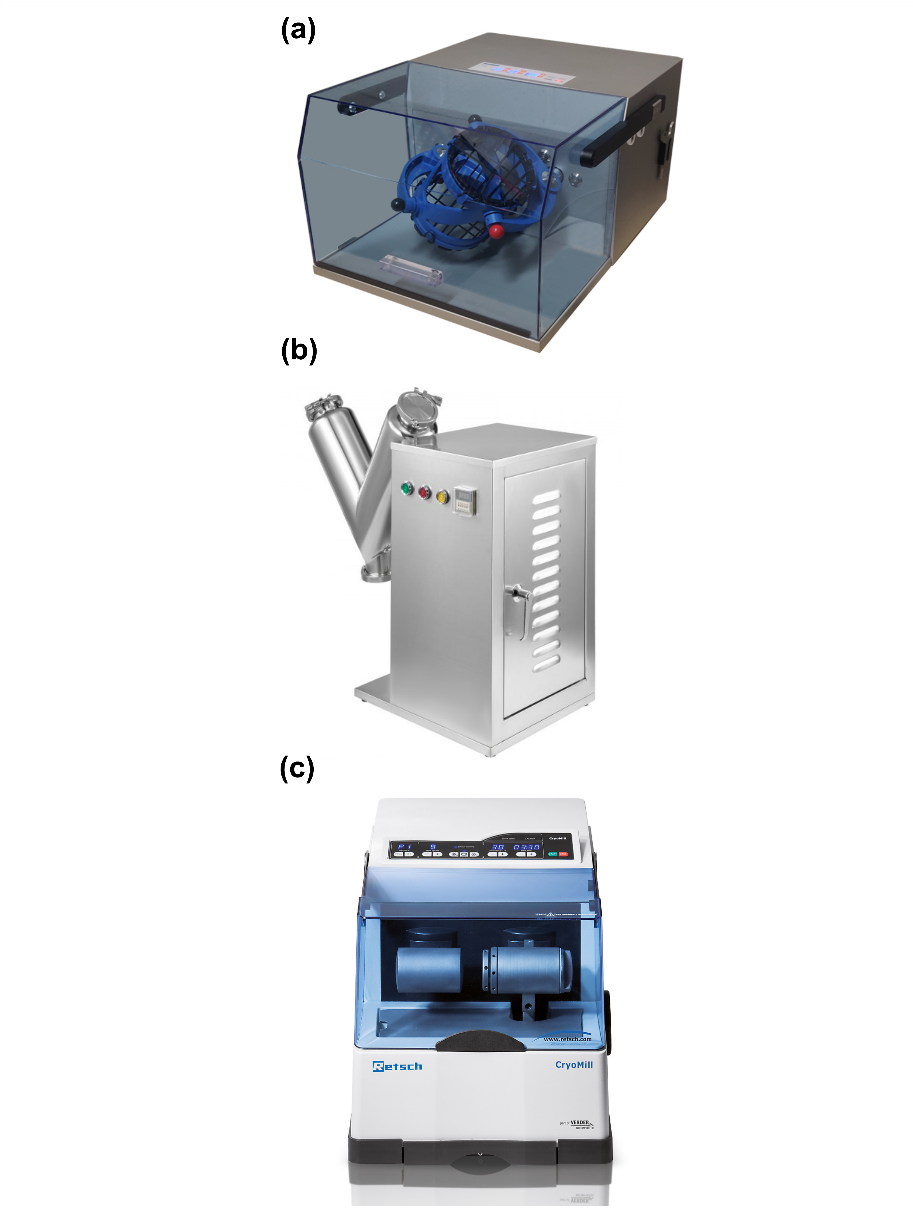


**Fig**. **1** Homogenization mixer instruments used for the preparation of the PP tablets: Tumble mixer (a) (LM-TM100, Laarmann, Roermond, Netherlands: <https://www.laarmann.eu/>), VM Lab mixer (b) (LFA machines, <https://www.lfatabletpresses.com/>) and Ball Mill (c) (Retsch® CryoMill 100-240V, 50/60Hz: <https://www.retsch.it/>).

**Table** 1 Sample preparation procedure with Tumble and VM Lab mixer

|  | PP (g) | Lactose (g) | PEG 6000 (g) |
| --- | --- | --- | --- |
| Mixing approach | 0.018 | 1.05 | 1.05 |
| Mixing time | 10 min (Tumble mixer)  30 min (VM Lab mixer) | | |
| 1^st^ dilution step | - | 10.5 | 10.5 |
| Mixing time | 10 min (Tumble mixer)  30 min (VM Lab mixer) | | |
| 2^nd^ dilution step | - | 19.2 | 19.2 |
| Mixing time | 10 min (Tumble mixer)  30 min (VM Lab mixer) | | |
| 3^rd^ dilution step | - | 19.2 | - |
| Mixing time | 10 min (Tumble mixer)  30 min (VM Lab mixer) | | |
| 4^th^ dilution step | - | 50.5 | - |
| Mixing time | 10 min (Tumble mixer)  30 min (VM Lab mixer) | | |
| 5^th^ dilution step | - | 104.0 | - |
| Mixing time | 20 min (Tumble mixer)  30 min (VM Lab mixer) | | |

**Table** 2 Sample preparation procedure with Ball Mill

|  | PP | Lactose | PEG 6000 |
| --- | --- | --- | --- |
|  | g | g | g |
| Mixing approach | 0.00072 | 8.6 | 1.3 |
| Mixing time | 10 min pre­cooling  6 cycles (2 min each)  2 min cooling down | | |

**Table 3** Particle size distribution of PP, lactose and PEG 6000, reported as mean (n = 3) particle size (standard deviation), with relative standard deviation (RSD, %).

| Tablet components | Condition | D_10_ (µm) | D_50_ (µm) | D_90_ (µm) |
| --- | --- | --- | --- | --- |
| Polypropylene | unsieved | 102.4 (2.8), RSD 2.7% | 230.6 (6.7), RSD 2.9% | 333.8 (4.9), RSD 1.5% |
|  | sieved | 23.0 (0.2), RSD 0.7% | 48.4 (0.1), RSD 0.1% | 70.1 (0.0), RSD 0.0% |
| Lactose | unsieved | 68.5 (0.5), RSD 0.7% | 149.1 (0.4), RSD 0.2% | 226.9 (1.4), RSD 0.6% |
|  | sieved | 36.9 (1.4), RSD 3.7% | 72.7 (1.5), RSD 2.1% | 112.2 (1.1), RSD 0.9% |
| PEG 6000 | unsieved | 16.4 (1.5), RSD 8.9% | 99.2 (11.4), RSD 11.4% | 265.7 (6.7), RSD 2.5% |
|  | sieved | 12.3 (0.3), RSD 2.4% | 44.2 (1.0), RSD 2.3% | 106.5 (2.2), RSD 2.1% |


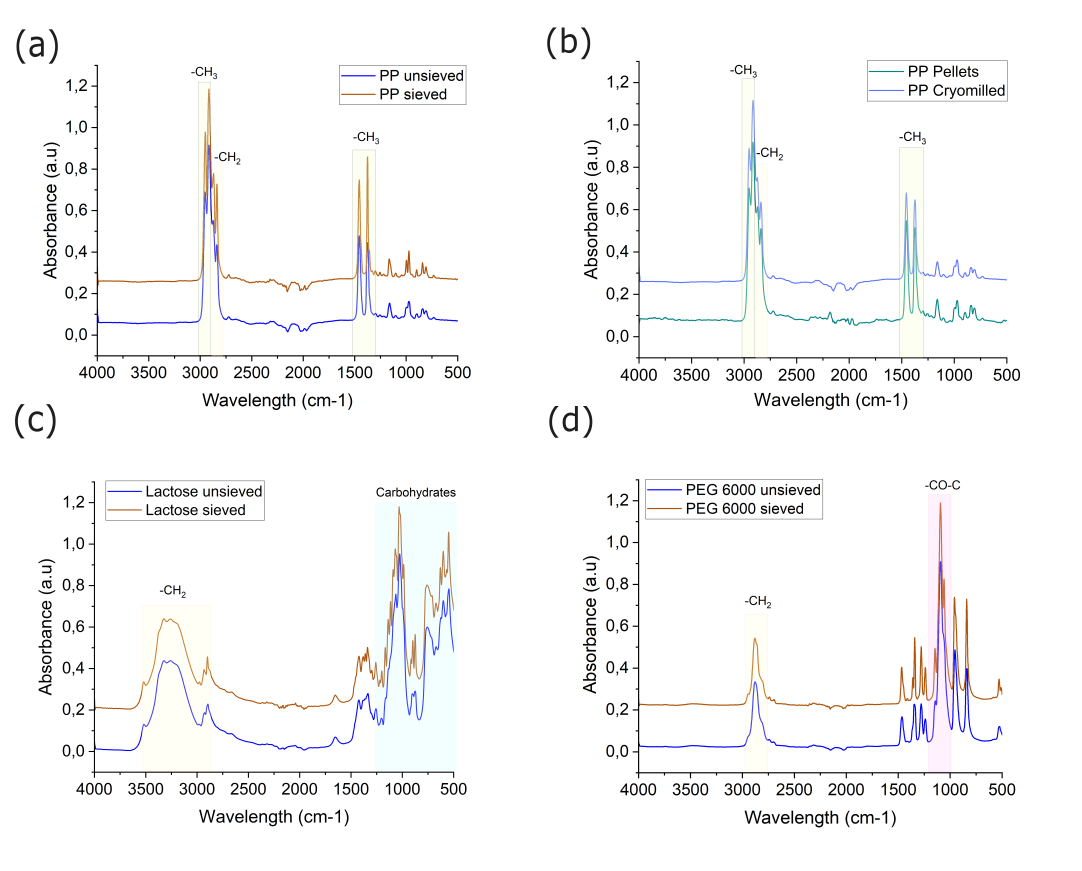


**Fig. 2** ATR-FTIR spectra of the three tablet component, sieved and unsieved: PP (a), lactose (c) and PEG 6000 (d). The analysis confirms that sieving procedure does not induce significant chemical or structural changes in any of the tablet components. In panel (b), the PP spectra before (pellet) and after (powder) cryomilling remain unchanged, confirming that the production process of PP does not alter its chemical or structure.


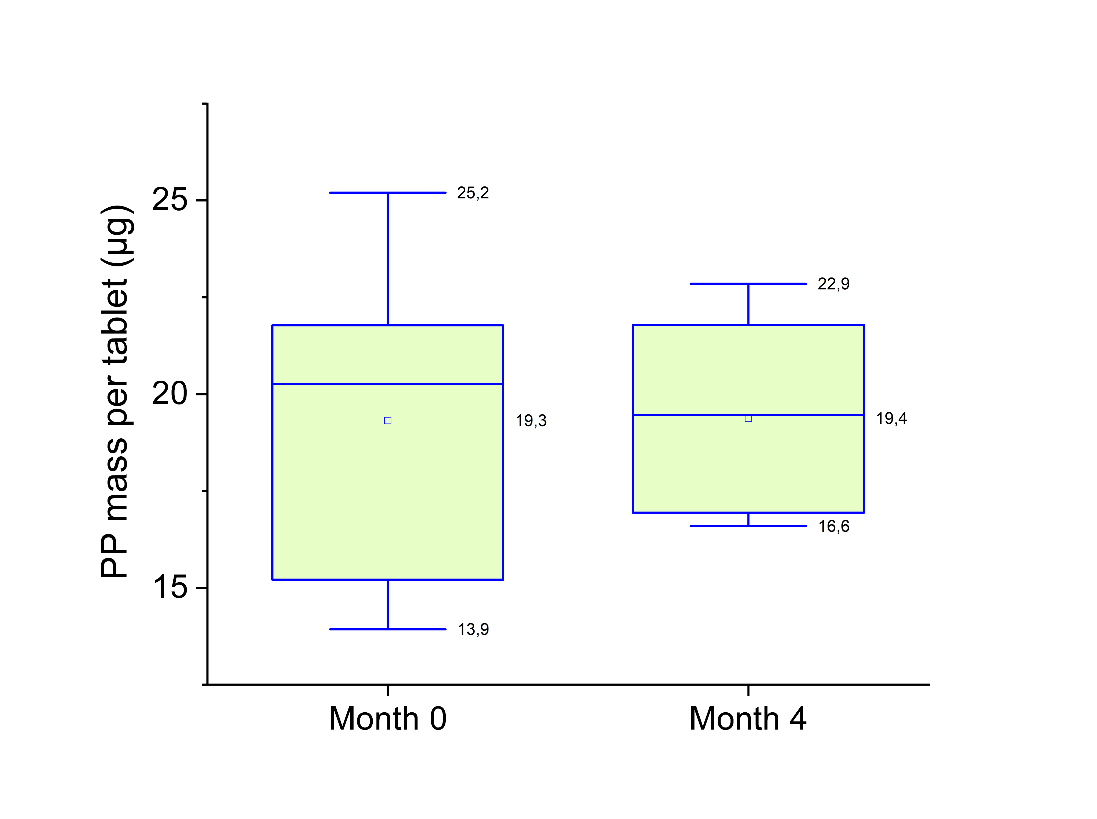


**Fig. 3** Distribution of PP mass over time for stability control assessment


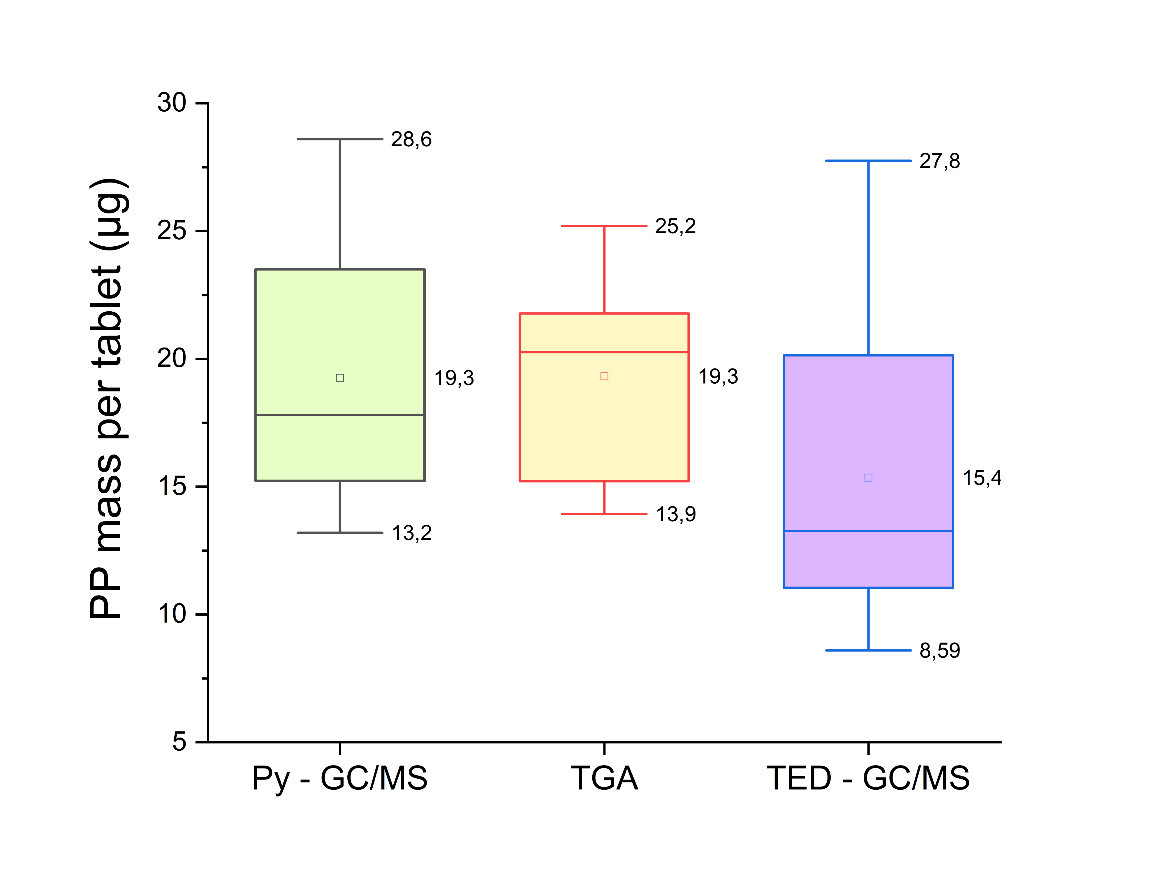


**Fig. 4** PP mass distribution for Sample 3 determined by mass-based methods (TGA, TED-GC/MS and Py-GC/MS) across two laboratories

**
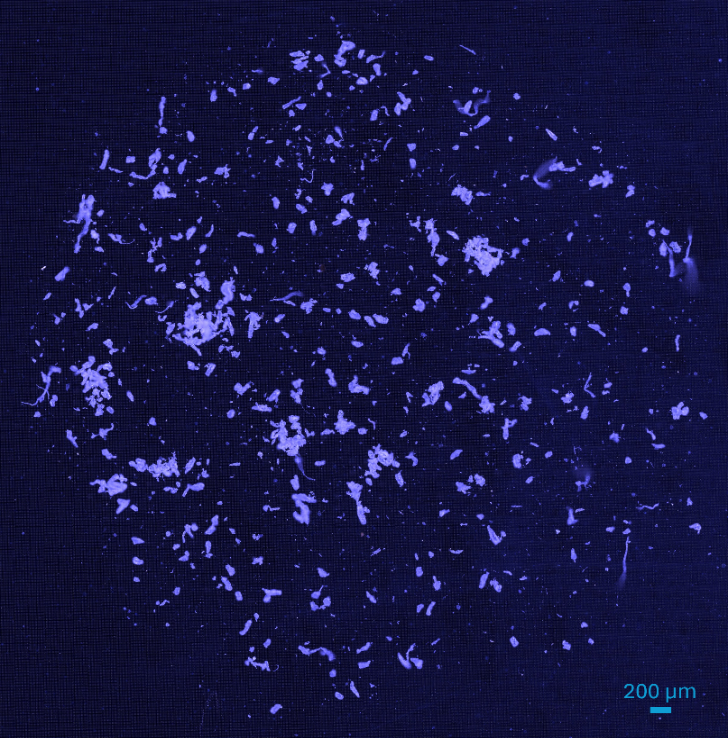
**

**Fig. 5** Distribution of PP MP particles on a Si filter after filtration

**Table 4** MPs particle number for each polymer type detected by µRaman analysis (n=10), reported as mean particle number (standard deviation).

| Polymers | Particle number |
| --- | --- |
| PS | 9 (7.0) |
| PP * | 1284 (240.0) * |
| PET | 15 (5.0) |
| PE | 1 (1.0) |
| PC | 0 |
| PVC | 0 |
| PMMA | 0 |
| PU | 0 |

*: PP MP particle number from the PP RM tablet

**Table 5** MPs particle number for each polymer type detected in procedural blanks by µRaman analysis (n = 3), reported as mean particle number (standard deviation).

| Polymers | Particle number |
| --- | --- |
| PS | 0 |
| PP | 3 (1.0) |
| PET | 15 (6.0) |
| PE | 0 |
| PC | 0 |
| PVC | 0 |
| PMMA | 0 |
| PU | 0 |
